# Reviewed article: Araujo MC, Passinho RS, Pereira RSF, Mora DJ. Temporal trends in incidence and mortality from pulmonary tuberculosis: time series study, Southern Bahia, Brazil, 2010-2023. Epidemiol Serv Saude. 2025:34;e20240778.

**DOI:** 10.1590/S2237-96222025v34e20240778.a

**Published:** 2025-08-04

**Authors:** Liandro da Cruz Lindner

**Table:** 

Rating	Excellent	Good	Average	Below average	Poor
Interest		✓			
Quality			✓		
Originality			✓		
Overall			✓		

**Table:** 

Questionnaire	Yes	No	Not applicable
Does the manuscript contain new and significant information to justify publication?	✓		
Does the Abstract (Summary) clearly and accurately describe the content of the article?		✓	
Is the problem significant and concisely stated?	✓		
Are the methods described comprehensively?		✓	
Are the interpretations and conclusions justified by the results?		✓	
Is adequate reference made to other work in the field?	✓		

## Manuscript Structure

Length of article is: Too short

Number of tables is: Adequate

Number of figures is: Adequate

Please state any conflict(s) of interest that you have in relation to the review of this paper (state “none” if this is not applicable): None

## Comments

Sugiro ampliar a discussão e não ficar somente na apresentação de dados.

A tentativa de se fazer uma discussão de gênero e TB é bem básica, e estereotipada, sugiro ampliar ou descartar.

A citação do caso de Taiwan é desnecessária no meu ponto de vista e a do Paraná precisa ser melhor contextualizada.

As desigualdades sociais e suas relação com causas de descontinuidade do tratamento e morte estão já descritas em estudos sobre custos catastróficos, sugiro incluir e ampliar esta visão.

A citada abordagem mulisetorial pode ser melhor elucidada.

A comparação com a Covid, na parte final. pode ser melhor explorada.

